# Hydrolyzation of mogrosides: Immobilized β‐glucosidase for mogrosides deglycosylation from Lo Han Kuo

**DOI:** 10.1002/fsn3.932

**Published:** 2019-01-29

**Authors:** Hsueh‐Ting Wang, Jin‐tong Yang, Kuan‐I Chen, Tan‐Ying Wang, Ting‐Jang Lu, Kuan‐Chen Cheng

**Affiliations:** ^1^ Graduate Institute of Food Science and Technology National Taiwan University Taipei Taiwan, ROC; ^2^ Institute of Biotechnology National Taiwan University Taipei Taiwan, ROC; ^3^ Department of Medical Research China Medical University Hospital China Medical University Taichung Taiwan, ROC

**Keywords:** glucosidase, immobilized enzyme, Lo Han Kuo, mogroside IIIE, mogroside V

## Abstract

An immobilized enzyme system for bioconversion of Lo Han Kuo (LHK) mogrosides was established. β‐Glucosidase which was covalently immobilized onto the glass spheres exhibited a significant bioconversion efficiency from pNPG to pnitrophenol over other carriers. Optimum operational pH and temperature were determined to be pH 4 and 30°C. Results of storage stability test demonstrated that the glass sphere enzyme immobilization system was capable of sustaining more than 80% residual activity until 50 days, and operation reusability was confirmed for at least 10 cycles. The Michaelis constant (*K*_m_) of the system was determined to be 0.33 mM. The kinetic parameters, rate constant (*K*) at which Mogrosides conversion was determined, the *τ*_50_ in which 50% of mogroside V deglycosylation/mogroside IIIE production was reached, and the *τ* complete of complete mogroside V deglycosylation/mogroside IIIE production, were 0.044/0.017 min^−1^, 15.6/41.1 min, and 60/120 min, respectively. Formation of the intermediates contributed to the kinetic differences between mogroside V deglycosylation and mogroside IIIE formation.

## 1 INTRODUCTION

The fruit of *Siraitia grosvenorii* Swingle, Lo Han Kuo (LHK), contains a mixture of curcurbitane‐type triterpene saponins, known as mogrosides. A mogroside is composed of several glycosylated sugar moieties which form β‐linkages with mogrols (Wang, Chiu, Lu, & Lo, 2018). *S. grosvenorii* fruit is widely used in Traditional Chinese medicine and was regarded as nontoxic sweetener. Its functions include moisturizing lungs and soothing coughs, reducing blood pressure, and preventing constipation (Pawar, Krynitsky, & Rader, 2013). Mogrosides have recently been classified as “generally recognized as safe” (GRAS) substances by the U.S. Food and Drug Administration (FDA) (GRAS Notice, 2017). Several studies have reported that mogroside extracts possess various biological activities, including antioxidative anti‐inflammatory and against diabetic (Liu, Wang, Qi, Zou, & Sun, 2018; Qi, Chen, Zhang, & Xie, 2008; Shi, Zheng, Wang, Liu, & Chen, 2014). In addition, the bioactivities of certain specific mogrosides have been reported. For example, mogroside V (MG‐V) (five glycine/glucose) attaching to a mogrol was shown to regulate insulin secretion in an in vitro cell model system (Zhou, Zheng, Ebersole, & Huang, 2009). Mogroside III (MG‐III) inhibited intestinal maltase and suppressed the rise in blood glucose after a single oral administration of maltose in rats (Suzuki, Murata, Inui, Sugiura, & Nakano, 2005). MG‐III reduced pulmonary fibrosis and may have therapeutic potential for treating fibrosis (Tao et al., 2017).

Several studies have been carried out to enhance the bioactive form of mogrosides, and the most common conversion method used is acid hydrolysis (Wang et al., 2018). LHK extracts were treated by hydrochloric acid, generating different type of aglycones (Chen et al., 2011). Although acid hydrolysis method can easily produce mogrosides at low cost, several potential drawbacks need to be overcome, such as poor specificity, low yield, and unexpected by‐products (Girisuta, Danon, Manurung, Janssen, & Heeres, 2008).

Enzymatic method has been used to producing mogrosides. For example, cellulase is able to convert mogroside V into mogroside III and mogroside II E (Murata et al., 2010); maltase (an α‐glucosidase) converts mogroside V into several types of mogroside (Takemoto, Arihara, Nakajima, & Okuhira, 1983). Compared to acid hydrolysis, enzymatic conversion method exhibits efficiency and specificity benefits. However, the cost of enzymatic conversion method is relatively high. Enzyme immobilization techniques provide several advantages, including easy separation of the enzyme from the reaction mixture, cost reduction by improving the reusability, continuous processing, and improvement of stability (Dervakos & Webb, 1991). In food industry, immobilized lactase catalytic system provides lactose‐free milk for patients with lactose intolerance; hydrolysis of glycosylated‐isoflavones of black soymilk using immobilized β‐glucosidase was also reported (Chen, Lo, Su, Chou, & Cheng, 2012; Chen et al., 2013; Ko et al., 2018).

In this study, we evaluated the feasibility of mogrosides conversion using an immobilized β‐glucosidase enzyme system. β‐Glucosidase was immobilized on three different carriers (glass spheres, nylon pellets, cellulose beads) for mogrosides deglycosylation, and *p*‐nitrophenyl β‐D‐glucuronide (*p*NPG) was employed as a chromogenic substrate to determine β‐glucosidase activity. The carrier possessing the most efficient catalytic behavior among these three solid carriers was chosen and applied to mogrosides deglycosylation for Lo Han Kuo extract solution. The kinetic parameters of the chosen catalytic system for mogrosides deglycosylation were further determined.

## 2 MATERIALS AND METHODS

### 2.1 Materials

Lo Han Kuo extract powder (Mogroside V ≥25.0%) was purchased from Huitong bio‐technology company (Guangxi, China). β‐Glucosidase from *Aspergillus niger* was purchased from Sigma‐Aldrich (St. Louis, MO). 4‐Nitrophenyl β‐D‐glucuronide (*p*NPG) purchased from Alfa Aesar (Ward Hill, MA) was used as the substrate to determine the catalytic activity of immobilized enzyme system. Glass spheres, nylon pellets, and cellulose beads were separately utilized as solid carriers for enzyme immobilization. Glass spheres were purchased from Sigma‐Aldrich with 9–13 μm particle size. Nylon pellets were provided by Keen Corp Company (Tainan, Taiwan). Porous cellulose beads were synthesized as previously described (Chen & Tsao, 1976). The particle size of nylon pellets and cellulose beads was about 1 mm.

### 2.2 Methods

#### 2.2.1 Carrier preparation and activation

##### Nylon pellets

Nylon pellets modification procedure has been reported previously (Diano et al., 2008).

In brief, a total volume of 40 cm^3^ nylon pellets was immersed in 30 ml of dimethyl sulfate reagent for 4 min at 100°C. A cleaning step was followed immediately by cold methanol, and then O‐alkylated nylon pellets were immersed in 50 ml of 10% hexamethylenediamine (HMDA) aqueous solution at room temperature for 90 min.

##### Cellulose beads

An organic solution (acetone/DMSO = 6:4) was used to dissolve cellulose acetate (6.25 g). The solution was then homogenized and added drop‐wisely into cold water by syringe, so that cellulose beads will precipitate and form (Chen & Tsao, 1976). Finally, the beads were washed with distilled water and dried overnight at room temperature to attain a constant weight. To enhance the hydroxyl groups on cellulose beads’ surface, a pretreatment was carried out in 0.2 M NaOH for 90 min.

##### Glass spheres

First, a total volume of 40 cm^3^ glass microspheres was incubated with a concentration of 10% nitric acid (HNO_3_) at 90°C for 1 hr. Next, microspheres were continuously washed with distilled water and were subsequently immersed in a 10% 3‐aminopropyltriethoxysilane (APES) aqueous solution (50 ml, pH 3.4) at 70°C for another 3 hr. Treated aminopropyl‐glass was cleaned with distilled water and dried overnight in an oven at 80°C. The procedure has been reported previously (Chen et al., 2012).

##### Enzyme immobilization

After preparation and activation, each activated carrier was treated with 2.5% glutaraldehyde aqueous solution. Cellulose beads were subjected to submersion at 100°C for 30 min, while nylon pellets and glass spheres were both treated under a gentler condition (room temperature, 60 min) (Chen & Tsao, 1977). The carrier was washed with double‐distilled water at room temperature, and each type of carrier was incubated separately with 20 mg of β‐glucosidase (dissolved in 50 ml of 0.1 M phosphate buffer at pH 6) at 4°C for 16 hr. Uncross‐linked enzymes were removed by the washing with 0.1 M phosphate buffer, and the enzyme‐linked carriers were stored at 4°C until use. The washing solutions were collected to detect the amount of uncrossed‐linked enzyme, β‐glucosidase. The amount of β‐glucosidase being successfully immobilized onto carrier was calculated as the difference between the initial and the uncross‐linked β‐glucosidase. Bradford dye‐binding method was adopted to determine the concentration of β‐glucosidase (Bradford, 1976). Five milligrams of *p*NPG was dissolved in 0.1 M phosphate buffer (50 ml, pH 4). Each type of enzyme immobilized beads was incubated in 50 ml of *p*NPG solution in a batch reactor at 30 and 50°C, respectively. A volume of 800 μl of *p*NPG solution was withdrawn, and 1 ml of 2 M sodium carbonate was added to terminate the reaction. The absorbance of *p*NPG was read at 425 nm and recorded at regular time intervals. All of the experimental studies were performed in triplicates.

##### Morphology characterization

The surface morphologies of glass spheres with and without β‐glucosidase immobilization were investigated using scanning electron microscopy (SEM) at an accelerating voltage of 10 kV (Model: JSM‐6300, JEOL; Tokyo, Japan). Prior to measurement, samples were coated with a thin layer of gold.

##### Verification of enzyme immobilization

The successful enzyme immobilization was confirmed by X‐ray photoelectron spectroscopy (XPS), also known as electron spectroscopy for chemical analysis (ESCA), a widely used surface analysis technique. ^16^ XPS measurements were carried out with a ESCA system (VG MICROTECH, MT‐500, British) using A1Ka radiation (hm = 1,486.6 eV); Thermo Scientific Avantage Data System software (Thermo Fisher Scientific Inc., Herts, UK) was utilized to perform curve fitting and calculate the atomic concentrations.

##### Mogrosides hydrolyzation, purification, and analysis

Hydrolyzation of Lo Han Kuo extract aqueous solution was performed with the free and immobilized enzymes. The hydrolyzation was carried out at pH 4.0, 30 or 50°C. Five hundred microliter solution of sample were withdrawn at regular intervals, and an equal volumes of methanol (100%) were added (into the sample) to terminate the reaction. To purify mogrosides from the above mixture solution, a separation was performed on a reversed‐phase C‐18 solid phase‐extraction cartridges (500 mg/3 ml, Chrome expert, Sacramento, CA, USA) to elute out (under 45%–80% of methanol) the impurities and collect different mogrosides in certain region. The collected sample was concentrated by vacuum evaporator and resolve in the 1 ml methanol which contained 40 ppm hydrocortisone (internal standard) solution.

To determine the kinetic parameters, the glucose detection in the reaction solution was accomplished by Megazyme D‐Glucose (glucose oxidase/peroxidase; GOPOD) assay kit (Wicklow, Ireland). Addition of 1 ml of GOPOD Reagent to 0.1 ml of sample solution followed by an incubation at 45°C for 20 min. The absorbance of each solution was then read at 510 nm.

##### Kinetic parameters

The Michaelis constant (*K*_m_) and maximal velocity (*V*_max_) for free and immobilized β‐glucosidase were determined at 30°C for 10 min. The Michaelis constant (*K*_m_) reflects the effective characteristics of the enzyme and the affinity between enzyme and substrate, which was determined using the Michaelis–Menten equation.

$$V=V_{\max}\left[S\right]/K_{m}+\left[S\right]$$

Here, [S] represents for various concentration of the substrate, pNPG. Maximal velocity (*V*_max_) stands for the maximum rate achieved by the enzymatic system, at saturating substrate concentration (The intrinsic characteristic of the enzyme, at which all the enzyme active sites are all bound to substrate.) The Michaelis constant (*K*_m_) is the substrate concentration where the reaction rate is half of *V*_max_.

##### HPLC‐UV analysis

Analysis Resolution of mogrosides was performed using Luna C18(2) 100A column (5 μm, 250 × 4.6 mm) and maintained at room temperature. The column was attached to a HPLC workstation containing two LDC pumps and a LDC analytical mixer. The injection volume was 20 μl with an elution rate at 0.6 ml/min. Detection was monitored at 210 nm, and the acquired data were analyzed by SISC chromatography data system (SISC, Taiwan).

##### Statistical analysis

All experiments, including carrier comparison, evaluation of reaction condition, absorbance measurements, and HPLC analysis, were performed in triplicates. For fraction analyses, data are presented as the means ± standard deviations. Differences among the variants were analyzed using one‐way analysis of variance with the Dunnett test; *p *<* *0.01 was considered statistically significant. We used a two‐sample *t* test (*F* < 0.001) to evaluate the effect of focus on bioactivity.

## 3 RESULTS AND DISCUSSION

### 3.1 Selection of carrier for immobilized enzyme system

**Figure 1:**
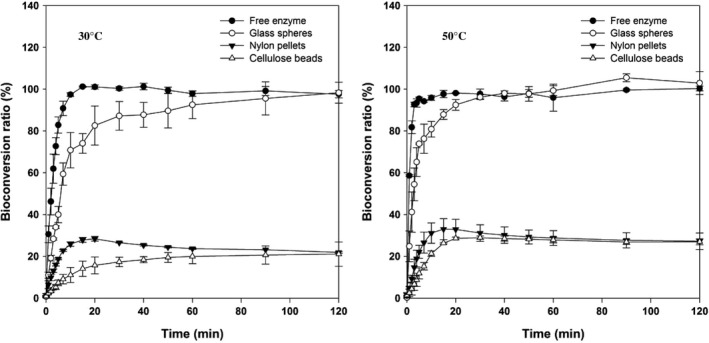
Catalytic behaviors of the suspended β‐glucosidase and the immobilized β‐glucosidase system using three different carriers (glass spheres, nylon pellets, cellulose beads) at 30 and 50°C. The relative activity was defined as the ratio of Conc.(t)/Conc.(s), where Conc.(t) represents the concentration of the *p*‐nitro‐phenol solution after t min of glucosidase system treatment and Conc.(s) represents the concentration of *p*‐nitro‐phenol converted completely from *p* NPG in the solution

To select a reliable immobilized enzyme system for mogrosides conversion, the immobilized enzyme content was calculated. The immobilized enzyme content of nylon pellets, cellulose beads, and glass spheres was 16.60, 3.52, and 18.00 mg, giving protein binding ratio at 83.0%, 17.6%, and 90.0%, respectively (Table 1). There was no significant difference in the amount of immobilized enzyme between nylon pellets and glass spheres. Figure 1 summarized the performance of three carrier and suspension enzyme systems at two temperatures (30 and 50°C) where *p*NPG was used as a substrate to measure enzyme activity. Results indicated that the immobilized β‐glucosidase on the glass sphere exhibited higher relative reactivity (30°C, 60 min, 93%; 50°C, 60 min, 99%) than those on the nylon pellets (30°C, 60 min, 24%; 50°C, 60 min, 29%) and cellulose beads (30°C, 60 min, 20%; 50°C, 60 min, 28%). The saturated conversion of *p*NPG to *p*NP by immobilized glass spheres enzyme system can be achieved within 30 min, and its catalytic effectiveness was close to the suspension enzyme system. The particle size is described as an important factor to catalytic activity for the immobilized enzyme system. System with smaller particles possesses higher catalytic activity due to larger specific area or minimized diffusion limitation (Alagöz, Tükel, & Yildirim, 2016; Mendes et al., 2012). The particle size of glass spheres used in this study was 9–13 μm, which is smaller than the nylon pellets and cellulose beads (about 1 mm). Therefore, glass spheres were selected as the immobilized carrier for the rest of study.

**Table 1:** Amount of the immobilized β‐glucosidase on the carriers

Carriers	Bound protein (mg)	Binding ratio (%)
Nylon pellets	16.60 ± 1.55^a^	83.0 ± 7.8^a^
Cellulose beads	3.52 ± 1.47^b^	17.6 ± 7.4^b^
Glass spheres	18.00 ± 2.07^a^	90.0 ± 10.4^a^

*Note*

Different letter superscripts in the same column are significantly different by Dunnett test (*p* < 0.01), *n* = 6.

### 3.2 Morphology of glass spheres

**Figure 2:**
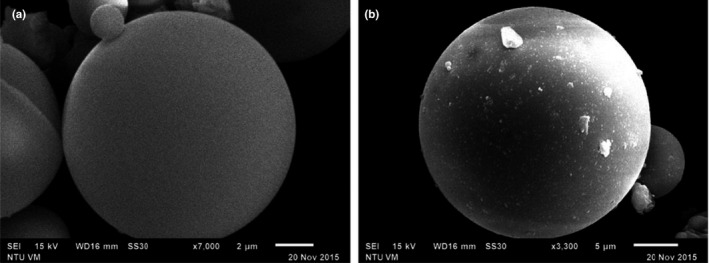
Glass spheres without (a) and with (b) β‐glucosidase immobilization

The surface morphologies of glass spheres with and without β‐glucosidase immobilization were investigated by scanning electronic microscope (SEM). Prior to the enzyme immobilization, the surface of the glass spheres was smooth (Figure 2a) and became granulated after enzyme immobilization (Figure 2b). This phenomenon echoes the observation of previous study (Chen et al., 2012; Ko et al., 2018). The granulated areas on the surface of glass spheres represented the regions of β‐glucosidase attachment (as pointed by arrows) providing evidence that enzyme was successfully immobilized onto the glass spheres.

### 3.3 Chemical modification of β‐glucosidase onto glass spheres

**Figure 3:**
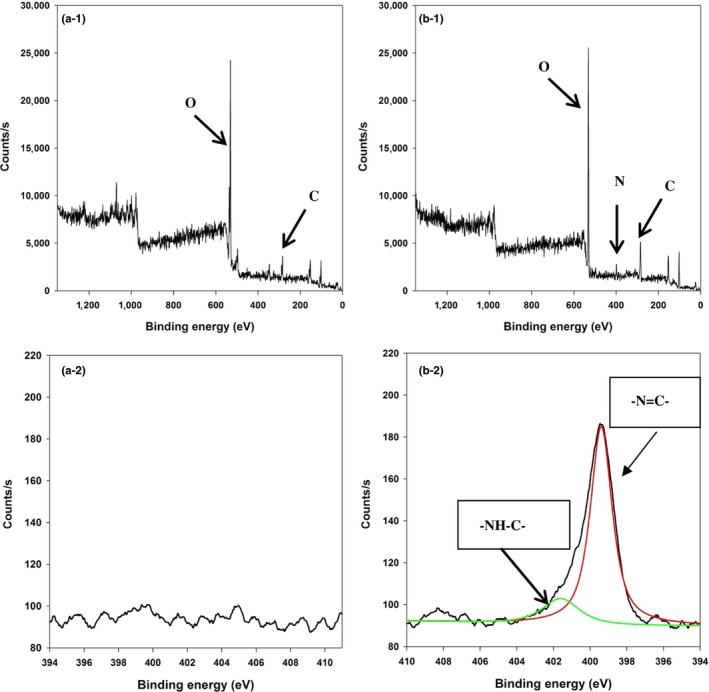
The observed full survey scan (1) and N 1s (2) ESCA spectra to examine the glucosidase immobilization: (a) without and (b) with β‐glucosidase immobilized on glass spheres. The characteristic elements and functional groups responsible for corresponding ESCA signals are indicated by arrows

β‐Glucosidase immobilized on the glass spheres was applied to hydrolyzation of mogrosides. When β‐glucosidase was immobilized on the carriers, ‐OH groups cross‐linked with glutaraldehyde through covalent binding. Subsequently, amino acid of β‐glucosidase reacted with glutaraldehyde by –C=N– bonds, which led to the attachment between enzymes and carriers. In this study, ESCA was used for elemental analysis and surface chemical characteristic analysis. Figure 3 demonstrated the characteristic elements of unmodified and modified glass spheres. Prior to enzyme immobilization, the full scan ESCA spectra (panel a‐1 in Figure 3) depicted the element characteristic signals at 284, 532 eV, and both 149 and 100 eV represented to the carbon (C), oxygen (O), and silicon (Si), respectively. Contrary, as illustrated in panel b‐1 (Figure 3), an extra signal was seen at 399 eV after enzyme immobilization, which corresponded to the signal of nitrogen element derived from β‐glucosidase. Moreover, in the N 1s ESCA spectra (panel a‐2 in Figure 3), no apparent peak was observed before enzyme immobilization, and two significant peaks at 399.8 eV (C=N) and 401.7 eV (C‐N) were observed in the N 1s ESCA spectra, which is in accordance to the previous study as the evidence of the nitrogen atom linkage (panel b‐2 in Figure 3) (Longo, Vasapollo, Guascito, & Malitesta, 2006). The peak at 399.8 eV in the N 1s ESCA spectrum can be verified that the amino acid of β‐glucosidase cross‐linked with glutaraldehyde through covalent binding (Chen et al., 2013). As a result, the successful enzyme immobilization onto glass spheres was confirmed.

### 3.4 Optimal pH and temperature for β‐glucosidase bioconversion

**Figure 4:**
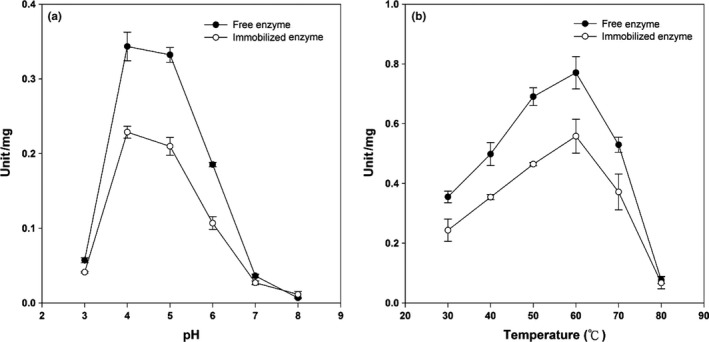
Effect of pH (a) and temperature (b) on the activity of suspension and immobilized enzyme systems. One Unit activity corresponds to the amount of enzyme which hydrolyzes 1 μmol *p*‐nitro‐phenyl‐β‐D‐glucopyranoside per minute

The enzyme activity is significantly affected by the reaction temperature and pH. β‐Glucosidase was stable in the acidic environment, and the optimum range of pH was between 4 and 5 (Kita et al., 1991; Vazquez‐Ortega, Alcaraz‐Fructuoso, Rojas‐Contreras, López‐Miranda, & Fernandez‐Lafuente, 2018). In this study, the efficiency of the immobilized enzyme system was evaluated and compared with the one from suspension enzyme at different pHs and temperatures. First of all, the enzyme activity was evaluated at different pH conditions at 30°C. It was noted in Figure 4a that the highest catalytic activity of both free and immobilized enzyme systems was reached at pH 4. Subsequently, evaluation of temperature on β‐glucosidase activity was carried out at pH 4. Figure 4b showed that β‐glucosidase exhibited the highest efficiency both in the free and immobilized enzyme systems at 60°C. On the other hand, a dramatic declining was observed in both enzyme systems with further increased temperature (from 0.77/60 to 0.08/80°C for suspension enzyme; from 0.56/60 to 0.06/80°C for immobilized enzyme). Higher temperatures result in proteins denaturation, which may explain the decrease in enzyme activity (Chen et al., 2012).

### 3.5 Determination of kinetic parameters for the enzyme systems

**Figure 5:**
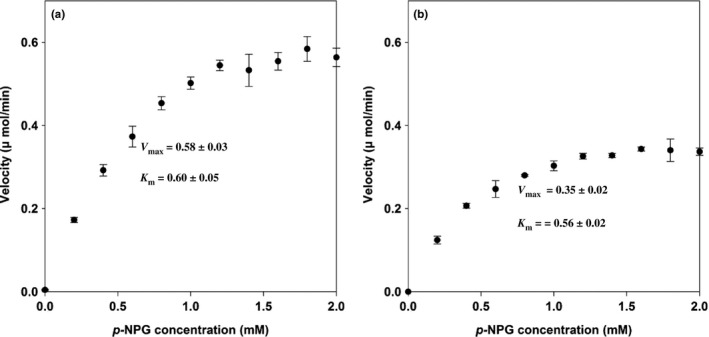
Effect of the concentration of *p* NPG on the activity of the suspension enzyme (a) and the immobilized enzyme (b). The reaction mixture was incubated at 30°C for 10 min. The velocity corresponds to the amount of *p*‐nitro‐phenyl hydrolyzed by enzyme per minute

In this study, *p*NPG was used to evaluate the hydrolyzation efficiency and to calculate enzyme kinetics. The kinetic parameters, *K*_m_ and *V*_max_, were 0.60 mM and 0.58 μmol/min for suspension enzyme and 0.56 mM and 0.35 μmol/min for immobilized enzyme system (Figure 5). Results indicated that the immobilized enzyme possessed similar affinity as the suspension enzyme did when saturated with pNPG. The *V*_max_ of immobilized enzyme system was approximately 40% lower than the value of suspension enzyme. This phenomenon maybe caused by the space steric hindrance which reduced the affinity between the enzyme and substrate, prolonging the reaction time (Talbert & Goddard, 2012).

### 3.6 Bioconversion of mogrosides

**Figure 6:**
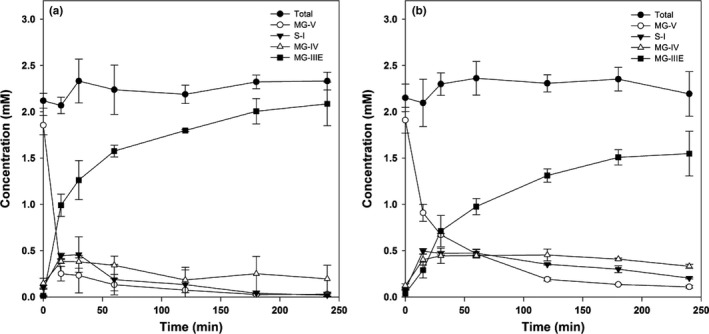
Bioconversion of mogrosides in Luo Han Kuo fruit extract solution by the suspension β‐glucosidase (a) and the immobilized glucosidase system (b). MG‐V, S‐I, MG‐IV and MG‐III represent Mogroside V, Siamenoside I, Mogroside IV, and Mogroside IIIE, respectively

**Figure 7:**
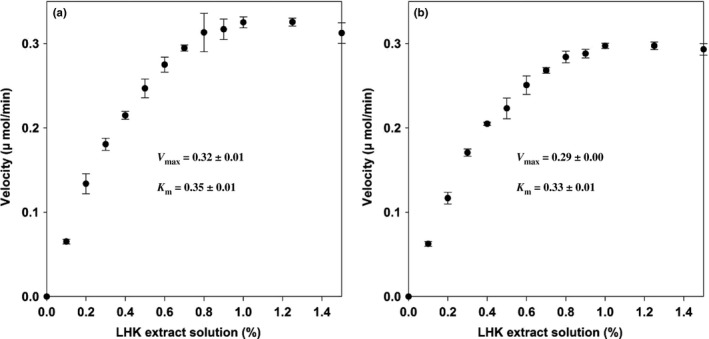
Determination of kinetic parameters for the free (a) and the immobilized (b) β‐glucosidase in different concentration LHK solution. The reaction mixture was incubated at 30°C for 30 min. The velocity corresponds to the amount of glucose released by glucosidase per minute

The hydrolyzation progress of mogrosides in luo han kuo fruit extract by free and immobilized β‐glucosidase systems can be seen in Figure 6. In both systems, the glycosylated mogroside (mogroside V) was firstly turned into intermediates (siamenoside I and mogroside IV) and eventually hydrolyzed into mogroside IIIE. The kinetic study of the free and immobilized enzymes for mogrosides conversion was done by calculating the amount of glucose released by β‐glucosidase (Figure 7). The *V*_max_ obtained from suspension enzyme and immobilized enzyme systems was 0.32 and 0.29 μmol/min. The *K*_m_ from the free and immobilized enzymes was 0.35 and 0.33 mM, respectively. The results demonstrated that the *V*_max_ of immobilized enzyme system was close to that of suspension enzyme, indicating the similar enzyme/substrate affinity. The kinetic parameters of mogrosides hydrolyzation were summarized in Table 2. It is obvious that mogroside V was quickly to be hydrolyzed in the suspension enzyme system since the *τ*_50_ was 4.6 min 5; however, it took 19.4 min to convert Mogroside V to Mogroside IIIE. Two intermediates, Siamenoside I and Mogroside IV, were formed during the hydrolyzation of Mogroside V (Chiu, Wang, Lee, Lo, & Lu, 2013). Mogroside V is the main compound extracted from Lo Han Kuo (LHK). However, different studies have been demonstrated that certain specific mogrosides exhibit different bioactivities (Chiu et al., 2013; Suzuki et al., 2005). Therefore, efforts have been made for mogrosides bioconversion. In the previous studies, microorganisms were used as bioconversion host for biotransformation of mogrosides (Chiu et al., 2013; Wang et al., 2015). Chiu et al. reported that Exg1 is a major enzyme for the initiation of mogroside V conversion and deletion of *KRE6* gene of *S. cerevisiae* facilitated mogroside IIIE production (Chiu et al., 2013). Wang et al. also reported a *Saccharomyces cerevisiae* mutant which can hyperproduction of β‐glucanase resulting in the enhanced bioconversion of mogrosides (Wang et al., 2015). However, it usually took more than 24 hr to reach complete mogrosides bioconversion. For our immobilized enzyme system, it took only 120 min to reach complete mogrosides bioconversion (Table 2). Moreover, the recovery and purification process is relatively easier for the samples from immobilized enzyme system than those from the fermentation ones.

**Table 2:** Kinetic parameters characterizing the suspension and the immobilized β‐glucosidase system

	Suspended β‐glucosidase		Immobilized β‐glucosidase	
	Mogroside V	Mogroside IIIE	Mogroside V	Mogroside IIIE
*K* (min^−1^)	0.149 ± 0.031	0.036 ± 0.006	0.044 ± 0.008	0.017 ± 0.002
*τ*_50_ (min)	4.6 ± 1.0	19.4 ± 3.1	15.6 ± 2.9	41.3 ± 3.9
*τ*_complete_ (min)	30	90	60	120

*Note*

Unpaired *t* test (*F* < 0.001) used to evaluate the effect of bioactivity, Mogroside V, and Mogroside IIIE was significantly different in conversion.

### 3.7 Reusability and storage stability

The reusable features of immobilized enzyme system enable the mogroside bioconversion for mass production. In this study, the immobilized enzyme system can be used consecutively for at least 10 cycles which still kept 90% of its original bioconversion activity (data not shown). A continuous bioconversion system can be adopted through bioreactor design (Ko et al., 2018). Although the conversion rate of suspension enzyme system is faster than that of immobilized enzyme system, the immobilized enzyme system exhibited advantages of recovery and purification. Through controlling the reaction time of immobilized enzyme system, moreover, we can manipulate the ratio of intermediates (Siamenoside I and Mogroside IV), and end product (Mogroside IIIE). Mogrosides are a group of triterpenoid glycosides found from the fruit of Lo Han Ko (*Siraitia grosvenorii*). A study reported that mogroside IIIE (with three glycosides) exerts greater anti‐hypoglycemic effect by inhibiting intestinal maltase activity than mogroside V (with five glycosides) (Suzuki et al., 2005). Besides, from an in vivo study, mogroside IIIE, a metabolite of Mogrosides, was widely distributed in the organ of treated rat (Chiu et al., 2013). Nevertheless, since the molecule structure is complex, it is difficult to synthesis or produce a specific type of mogrosides.

**Figure 8:**
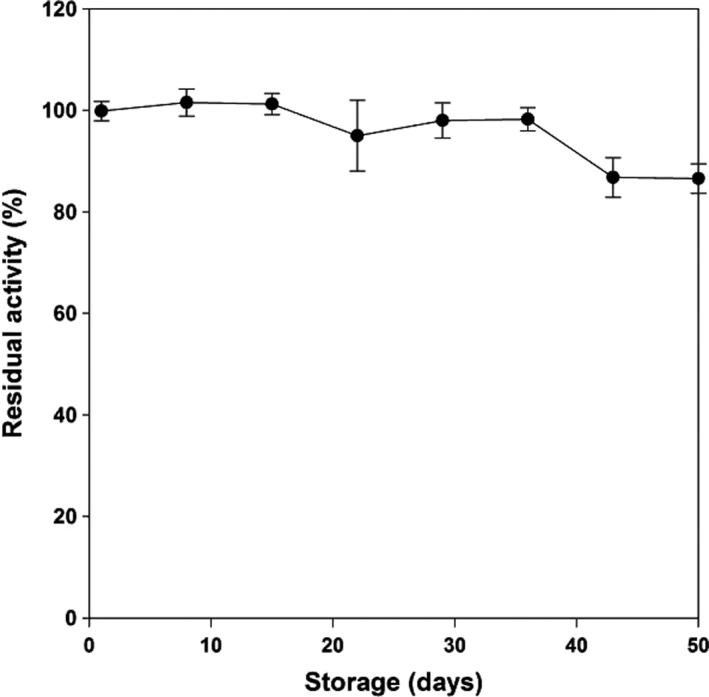
Storage stability of immobilized β‐glucosidase from 1 to 50 days

The storage stability of glass spheres catalytic system was also evaluated. As shown in Figure 8, more than 80% catalytic activity was retained up to 50 days. These results demonstrated that glass spheres catalytic system allowed a long‐period operation and was capable of retaining its enzyme activity.

## 4 CONCLUSION

In this study, we proposed the program by controlling reaction time to produce a specific type of mogroside, mogroside IIIE. The glass spheres catalytic system where β‐glucosidase was immobilized successfully converted mogroside V to mogroside IIIE. A reliable stability and reusability of the system was also reported. To our knowledge, this is the first time that the immobilized enzymes system has been applied to Lo Han Ko mogrosides hydrolyzation, which offers an alternative solution to specifically produce mogroside IIIE and, meanwhile, lowers the enzyme cost. Further study is still needed to improve the productivity of the system for practical applications. Different reaction profiles to produce specific mogrosides with different functions may be another task.

## CONFLICT OF INTEREST

The authors declared no conflicts of interest.

## ETHICAL STATEMENT

The current study was not required to complete an ethical assessment.
